# Tumour microenvironment influences response to treatment in oesophageal adenocarcinoma

**DOI:** 10.3389/fimmu.2023.1330635

**Published:** 2023-12-13

**Authors:** Clemence J. Belle, James M. Lonie, Sandra Brosda, Andrew P. Barbour

**Affiliations:** ^1^ Surgical Oncology Group, Frazer Institute, The University of Queensland, Brisbane, QLD, Australia; ^2^ Department of Surgery, Princess Alexandra Hospital, Brisbane, QLD, Australia

**Keywords:** oesophageal adenocarcinoma, tumour microenvironment, immune infiltrate, immunotherapy, treatment response, immune checkpoints

## Abstract

The poor treatment response of oesophageal adenocarcinoma (OAC) leads to low survival rates. Its increasing incidence makes finding more effective treatment a priority. Recent treatment improvements can be attributed to the inclusion of the tumour microenvironment (TME) and immune infiltrates in treatment decisions. OAC TME is largely immunosuppressed and reflects treatment resistance as patients with inflamed TME have better outcomes. Priming the tumour with the appropriate neoadjuvant chemoradiotherapy treatment could lead to higher immune infiltrations and higher expression of immune checkpoints, such as PD-1/PDL-1, CTLA4 or emerging new targets: LAG-3, TIM-3, TIGIT or ICOS. Multiple trials support the addition of immune checkpoint inhibitors to the current standard of care. However, results vary, supporting the need for better response biomarkers based on TME composition. This review explores what is known about OAC TME, the clinical significance of the various cell populations infiltrating it and the emerging therapeutical combination with a focus on immune checkpoints inhibitors.

## 1 Introduction

Oesophageal cancer is the sixth most lethal cancer worldwide (1). Although most cases are oesophageal squamous cell carcinoma (OSCC), its incidence is decreasing while the incidence of oesophageal adenocarcinoma (OAC) is rapidly rising worldwide (2). OAC cases outnumber OSCC in developed countries, which can be attributed to epidemiological risk factors. Known risks factors include increasing age, male sex, obesity, gastro-oesophageal reflux disease (GORD), smoking, and diets low in fibre (3). Over time GORD can lead to Barrett’s oesophagus (BO), a precancerous lesion with intestinal and gastric metaplasia composed of columnar and goblets cells replacing the normal squamous epithelia in the oesophagus. Symptoms appear at late stages, leading to late-diagnosis and poor survival. OAC 5-year survival is amongst the lowest (4). Perioperative chemotherapy or neoadjuvant chemo(radio)therapy (NAC) with surgery remains the standard of care for curative intent disease. However, with the advent of immunotherapy therapeutic options have expanded to include immune checkpoint inhibitors (ICI) in both the curative and palliative settings (5, 6). Significant treatment improvement has been made in other cancer types, but ICI response in OAC is moderate. Only a minority of patients shows a complete or partial response when treated with immunotherapy (7). Treatment strategies across cancers are moving towards personalised medicine. Hence, it is crucial to stratify patients and identify biomarkers to predict treatment efficacy and minimize toxicity.

Immunotherapy efficiency relies on the patient’s immune system, and more specifically on the immune cells surrounding and infiltrating the tumour. This immune infiltration, together with other cell populations, composes the tumour micro-environment (TME). Due to its potential predictive and prognostic value, the TME is becoming a major research focus to better tailor therapeutic strategies.

This review will explore the current knowledge concerning the OAC TME and focus on the clinical importance of considering the TME to guide therapeutic approaches.

## 2 OAC TME

Cancer is a genetic disease underpinned by DNA mutations that allow unregulated cell growth. These DNA mutations can result in the production of abnormal proteins, known as tumour antigens, which are eliminated by the host immune system in a process known as immunosurveillance. Tumours may escape immunosurveillance through several active mechanisms that results in the recruitment and modulation of diverse cell populations within the TME including immune and stromal cells. Therefore, characterising the TME in OAC is crucial to enabling informed patient selection to predict treatment response to standard therapies and ICIs and ultimately improve patient outcomes (8).

### 2.1 TME composition and clinical significance

Tumours evolve to form a complex ecosystem composed of cancerous cells infiltrated by diverse immune cells, mainly T cells, B cells and macrophages, and a rich stroma, mainly constituted of cancer-associated fibroblasts (CAFs), endothelial cells forming blood vessels and extracellular matrix components (9, 10). Depending on the proportion, location, and phenotype of the different cell populations, the TME switches between being pro- or anti-inflammatory (11). The TME communicates with tumour cells through cytokines, chemokines, and growth factors, leading to a dynamic tumour evolution following the environmental cues.

Chronic exposure of the oesophagus to gastric acid reflux favours a chronically inflamed environment. Recent studies showed a progressive shift in the immune populations present in the oesophagus during the evolution from BO to OAC (12, 13). Immune populations progressively invade BO microenvironment, however a diminution of the immune infiltrate is noted between a highly dysplastic oesophagus and OAC. The profiles of released cytokines and chemokines also support a more inflamed environment in OAC while immune-stimulating cytokines only show slight increase during the evolution from BO to OAC. Some evidence support the important role of CAFs in the development of BO and progression to OAC (14, 15). These immune changes contribute to the establishment of a pro-tumorigenic environment promoting OAC development (16).

**Figure 1:**
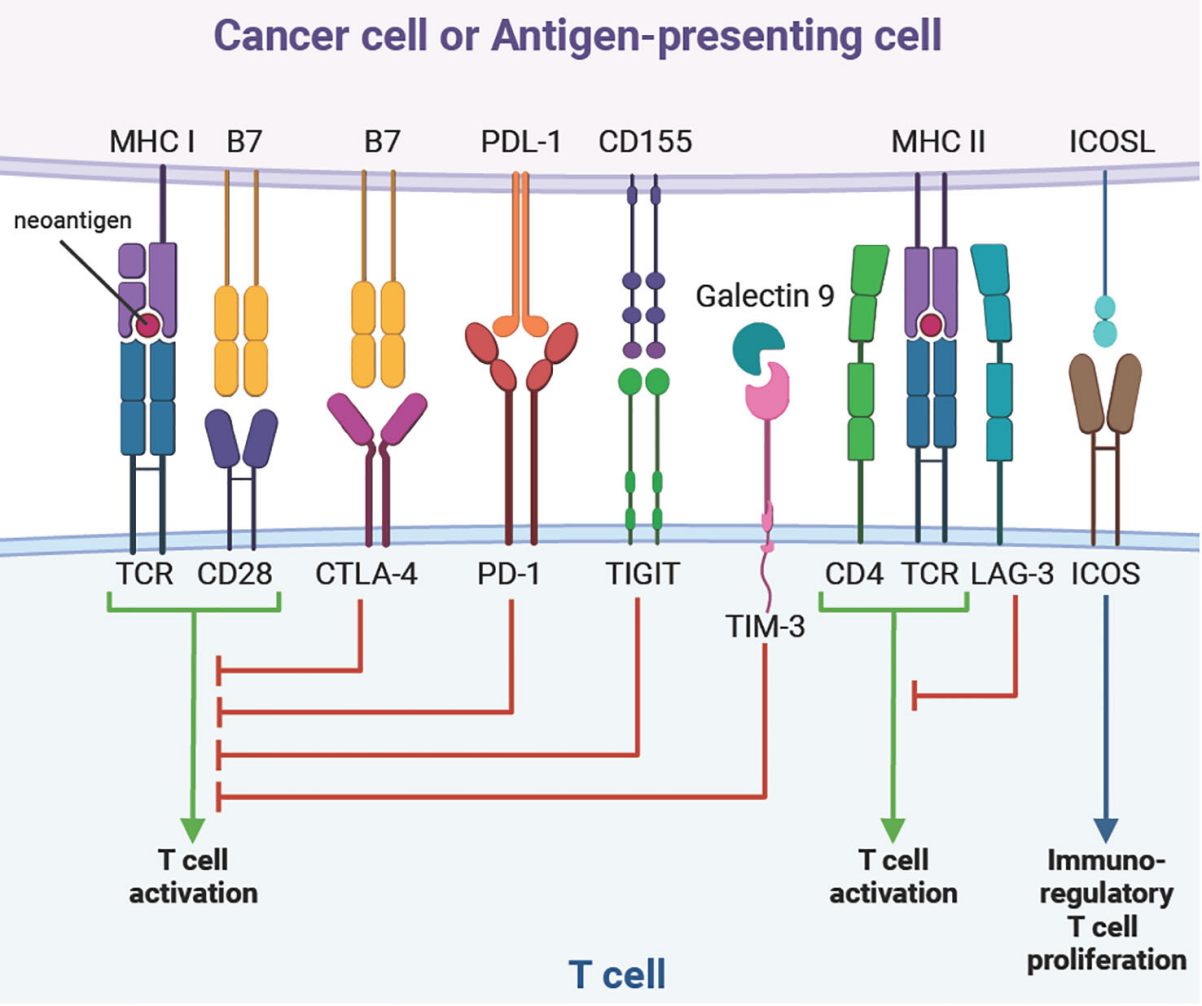
T cell immune checkpoints.

Several studies describing the TME in OAC associated clinical outcomes to the presence or absence of specific cell populations. It is widely accepted that a higher level of total immune cells, or total tumour-infiltrating lymphocytes, is linked to longer overall survival (OS) independently from the chosen therapeutic approach (17–22). Derks et al. reported OAC had a lower T cell density compared to gastric tumours (23). It is important to distinguish between phenotypes of the infiltrating T cell subpopulation. Higher immunoregulatory T cell infiltrations have been found in non-responders to NAC, whereas complete responders showed higher tissue-resident or circulating memory T cells (24, 25). Stein et al. found contradictory results with a positive correlation between high regulatory T cell infiltration and OS (26). Effector T cells can also become ineffective or differentiate to an immunoregulatory phenotypes with the overexpression of specific surface proteins, known as immune checkpoints. These include programmed cell death protein 1 (PD-1), cytotoxic T lymphocyte-associated protein 4 (CTLA-4), lymphocyte-activation gene 3 (LAG-3), T-cell immunoglobulin and mucin domain 3 (TIM-3) or inducible T-cell costimulator (ICOS) (**Figure 1**). Several studies linked the high expression of immune checkpoints with treatment response or OS (22, 27). The location of the immune infiltrate is also a key parameter. Patients with a higher T cell infiltration in the tumour core had a better response to NAC and a significant survival benefit (19, 25, 26). However, this benefit was not observed in patients with high T cell infiltration in the tumour invasive margin or in the tumour periphery. Other studies reported CD8+ T cells located in the stroma were also a prognostic marker associated with better OS (21, 22). This emphasises the importance to precisely characterise T cell infiltration with subpopulation phenotyping while assessing the spatial distribution of these cells.

Other immune populations play a role in cancer progression and treatment, but fewer studies described them in OAC. Haddad et al. reported an enrichment of macrophages with an anti-inflammatory phenotype in the TME (21). High anti-inflammatory macrophage infiltration has been linked to NAC non-responders and poor survival in small OAC cohorts (24, 28). Derks et al. reported that contrary to CD8+ T cells being included from the tumour core, macrophages seemed to be able to infiltrate it quite homogeneously (23). Mylod et al. reported low levels of infiltrating NK cells compared to circulating NK cells (29).

Emerging evidence suggests that stromal cells play an important role. Courrech Staal et al. validated a stroma score in OAC biopsies, a higher score was associated with better survival (30). Huai et al. found that OAC stromal cell infiltration was correlated with tumour stage (31). Activated CAFs correlated with poor OS and favoured OAC growth and invasion through paracrine communication (32). Sharpe et al. reported CAFs drive resistance to chemotherapy and found that blocking CAFs phosphodiesterase type 5 would prevent normal fibroblasts to differentiate into CAFs (33). Manousopoulou et al. found many differentially expressed genes between normal fibroblasts and CAFs in OAC tumours, supporting the pro-oncogenic phenotype shift happening during stroma remodelling (34). Moreover, the stroma is the environment favouring angiogenesis, a crucial phenomenon for cancer oxygenation, growth and later invasion to distant sites (10). Endothelial cells appeared to be an indicator of good pathological response to chemotherapy (35).

Lymph node and distant site metastases worsen the prognosis. Lower levels of immune cells, specifically CD8+ T cells, have been associated with higher lymph node invasion (17, 18, 21, 36). Macrophages tend to have an immunoregulatory phenotype in nodal-spread OAC (28). Dos Santos Cunha et al. also observed mast cells and NK cells in metastasised OAC were favourable prognostic factors. These findings support the hypothesis that immune surveillance failure participates in cancer invasion.

### 2.2 Immune classification of the TME and clinical significance

First initiated by Galon et al., the Immunoscore was described for colorectal cancer and was validated as a reliable biomarker of immunotherapy response (37, 38). The use of the Immunoscore has been extended beyond colorectal cancer and appears to be a strong prognostic factor for other solid cancer types, including OAC (39–41). The initial Immunoscore relied on the density of CD8+ effectors and CD3+CD45RO+ memory T cells presence in the tumour core and invasive margin. The Immunoscore was established as a prognostic factor for OSCC (42). However, when applied to OAC, Conroy et al. noticed a higher expression of CD8+ and CD45RO+ T cells in the stromal compared to the tumoural regions and failed to demonstrate an association with survival (16). Patient classification in different immune groups can be tailored for a specific cancer type by considering additional immune populations associated with treatment response and survival. It appears that OAC immune stratification requires further markers.

Recent studies focused on defining OAC immune groups. Naeini et al. identified four immune clusters based on proportions of immune cells in the TME (8). The immune hot group was enriched in immune cell infiltrate and associated with the best OS, while the immune suppressed group was enriched in macrophages but depleted of lymphocytes and associated with the worst OS. The immune moderate group with moderate levels of lymphocytes and depleted of other immune cells and the immune cold group lacking all immune cells showed moderate OS. More studies found several immune clusters in OAC patients and support that inflamed TME correlated with better outcomes (41, 43).

Establishing an OAC-specific immune score chart combining several immune features of the TME would be highly informative to predict treatment response and adjust the regimen administered to patients (44).

## 3 Treatment evolution in OAC and modulation of the TME

### 3.1 First generation of treatment for OAC and its impact on the TME

The TME is dynamic and evolves with tumour progression or via administration of cytotoxic therapies. Few studies have investigated the effect of cytotoxic therapies on the OAC TME. Understanding how both chemotherapy and radiotherapy regimens effect the different TME profiles of OAC will allow better treatment selection based on the patient’s TME profile. Previous studies have found no difference in infiltrating T cell subpopulations between patients who underwent different NAC treatments or surgery alone (18, 21, 43). However, Soeratram et al. found an increase of CD8+ T cell density in inflamed post-NAC tumours (22). Furthermore, two recent studies found a higher T cell infiltrate after therapy where there was a poor pathological response. Croft et al. performed a single cell analysis comparing treatment-naive and NAC-treated OAC samples (35). The most notable proportion changes in the TME profiles were a reduction of the NK and T cell populations, and an increase of B cells, endothelial cells and fibroblast populations. However, poor pathological responders kept a higher proportion of NK and T cells compared to good responders. This result was supported by Koemans et al. showing that pathological non-responders had a higher CD8+ T cell infiltration which was associated with worse OS, whilst no association between T cell infiltration and survival could be found for good and moderate responders (45).

Fewer studies investigated other components of OAC TME following NAC. Cao et al. noticed the correlation between macrophage pro- and anti-inflammatory phenotype ratio and survival is diminished after therapy (28). NAC appears to also greatly impact CAFs based on the high number of differentially expressed genes identified by Croft et al. between pre- and post-treatment samples (35).

NAC also appears to influence immune checkpoint expression. Conflicting results support the complexity of OAC response to treatment. Several studies found immune checkpoints, such as PD-1, CTLA-4, TIGIT, TIM-3, LAG-3 or ICOS, to be significantly upregulated in infiltrating T cells following NAC (46, 47). An OAC cell line study validated *ex-vivo* also suggested increased expression of several immune checkpoint expression following radiotherapy (48). Soeratram et al. found increased stromal PD-L1+ T cells after NAC, suggesting that PD-1/PD-L1 blockade may be the recommended therapeutic strategy following chemoradiotherapy (22). In contrast, Galvin et al. demonstrated a significant reduction of intra-tumoural PD-1 expression following NAC (49).

These findings on OAC TME modulation combined with previous evidence in other cancer types, support the potential role of chemo(radio)therapy in switching cold tumours to hot through triggering an immune response from dying tumour cells or new neoantigen generation (46, 50). This could prime tumours to be more responsive to immunotherapy.

### 3.2 Immunotherapy landscape in OAC

ICI is now an established therapy aimed at preventing the inhibition of the anti-tumour immune response (44). The earliest trials investigating ICIs in OAC focused on treating advanced/metastatic oesophageal or gastro-oesophageal cancers with anti-PD-1, or anti-PD-L1 monoclonal antibodies (6, 51, 52) and have been summarised in a previous review (7). Results varied, but overall, in the advanced/palliative setting ICI appears to improve patient survival compared to chemotherapy alone. CheckMate-577 is the largest adjuvant phase III study to date and investigated adjuvant nivolumab in patients with residual disease post CRT and surgery (6). Although disease-free survival rates were improved in OAC patients, pathological response was poor. Furthermore, various trials have incorporated PD-1 blockade in different lines of therapy and have found that patients expressing high levels of PD-1 have improved outcomes (NCT04802876) (53). These results suggest that an immune inflamed TME in OAC is necessary to derive benefit from immunotherapy. Thus, more trials incorporated PD-1 blockade in therapeutic protocols, even in the neoadjuvant or adjuvant setting (Keynote-585 (NCT03221426), NCT02918162) (54, 55). In the neoadjuvant setting results are conflicting.

Several trials investigated the feasibility of combining chemotherapy with anti-PD-L1 antibodies. Gemstone-303 (NCT03802591) noted a modest but significant improvement in OS and PFS when patients underwent PD-L1 blockade combined with chemotherapy (56). MATTERHORN (NCT04592913) reports more patients with a pathological complete response when anti-PD-L1 antibodies were added to chemotherapy compared to chemotherapy alone (57). DANTE (NCT03421288) showed beneficial effects of the addition of PD-L1 blockade to chemotherapy on pathological regression, especially for patients with higher PD-L1 expression (58). Finally, the addition of anti-PD-L1 antibodies did not increase the number of responders in the PERFECT trial (NCT03087864) (59). In the PERFECT trial, a biological sub-study was performed and suggested TME features could be used as response biomarkers (59). Good pathological responders had low expression of genes linked to ICI resistance and were found to have higher OS and PFS after PD-L1 blockade compared to chemotherapy-alone in good responders. Focusing on the non-responders, two subgroups were identified, with either high infiltration of CD8+ T cells presenting an exhausted phenotypes or with very low CD8+ T cell levels. These results were found in a small patient cohort (n=40) but showed the role of the TME in ICI response. Arbore et al. also reported a subset of CD8+ T cells were correlated with a better response rate to PD-1 blockade, confirming the potential of TME features as predictors of ICI response (24).

Concerning other major immune checkpoint of interest, CTLA-4, a phase-II trial (NCT01585987) completed in 2015 compared the efficacy of ipilimumab to the best standard of care for advanced gastro-oesophageal junction cancers. However, the results did not show benefit in OS or PFS for patients treated with ipilimumab (60). Since, the administration of anti-CTLA-4 antibodies has been investigated in combination with anti-PD-1 or anti-PD-L1 therapies. Checkmate-649 (NCT02872116) did not demonstrate better survival with nivolumab/ipilimumab combination (61). However, Checkmate-648 used the same treatment combination on OSCC patients and found improved OS compared to chemotherapy alone (62). A combination of durvalumab and tremelimumab treatment showed encouraging results in a small cohort of patients (n=114) by a phase I/II trial (NCT02340975) (63). However, targeting CTLA-4 does not appear to be the most beneficial approach for OAC patients.

A recent trial (NCT05187338) is looking at a triplex checkpoint inhibitor combination therapy with ipilimumab, pembrolizumab and durvalumab for a range of solid tumours. It will surely be interesting to compare how patients with oesophageal cancer respond to this combination therapy and the range of adverse effects.

The PERFECT study showed high number of CD8+ T cells with a high expression of exhaustion markers, such as PD-1, TIM-3 or LAG-3 in non-responders (59). This suggests that patients responding moderately or not at all to the current immunotherapy options may benefit from a new generation of immunotherapies, targeting different immune checkpoints, such as TIM-3, LAG-3, TIGIT or ICOS (16, 44, 64). Trials focussing on dual blockade combinations (LAG-3/PD-1, TIM-3/PD-1 or TIGIT/CTLA-4) are still at early stages of completion and feasibility, tolerability and safety exploration (**Table 1**). These mainly phase I or II trials represent the first step to a better understanding of TME response to these immune checkpoints in oesophageal cancers.

**Table 1:** Selected immunotherapy trials in Oesophageal cancers.

Clinical Trial Identifier	Phase	Completion date	Indications (population, N)	Arms	Target	Sponsor
NCT03044613	I	January, 2024	Gastroesophageal cancer - stage II/III, N=32	(A) Nivolumab + Relatlimab prior surgery (B) Nivolumab prior to neoadjuvant chemoradiation and surgery	LAG-3/PD-1	Bristol-Myers Squibb
NCT05342636	I/II	November 8, 2024	Advanced OSCC failed 1 line of therapy without prior PD1/PDL1 treatment, N=120	(A) Prembrolizumab + chemotherapy (B) Favezelimab/Pembrolizumab + chemotherapy (C) MK-4380 (anti-ILT4)/Pembrolizumab + chemotherapy	LAG-3/PD-1	Merck
NCT04785820	II	June 30, 2025	Advanced or Metastatic OSCC, N=210	(A) Lomvastomig (dual anti-TIM-3/PD-1) (B) Tobemstomig (dual anti-LAG-3/PD-1) (C) Nivolumab (anti -PD-1)	TIM-3/PD-1 and LAG-3/PD-1	Hoffmann-La Roche
NCT03708328	I	April 30, 2024	OSCC, N=134	Dose escalation of Lomvastomig	TIM-3/PD-1	Hoffmann-La Roche
NCT03652077	I	August 18, 2021	Various solid tumours (including Oesophageal cancer), N=40	Dose escalation of INCAGN02390	TIM-3	Incyte Corporation
NCT05834543	I/II	June, 2024	Advanced OSCC, N=75	(A) TQB2618 injection (anti-TIM3) + Penpulimab injection (anti-PD1) + chemotherapy (B) Penpulimab injection + chemotherapy (C) TQB2618 injection + Penpulimab + TQB3617capsules	TIM-3/PD-1	Chia Tai Tianqing Pharmaceutical Group Co., Ltd.
NCT04540211	III	February 13, 2023	Unresectable locally advanced, unresectable recurrent or metastatic Oesophageal cancer, N=461	(A) Atezolizumba + Tiragolumab + chemotherapy (B) Atezolizumab + chemotherapy	TIGIT/CTLA-4	Hoffmann-La Roche
NCT03281369	I/II	August 24, 2024	Locally advanced unresectable or metastatic G/GEJ cancer, N=410	(A) Atezolizumab + Tiragolumab + chemotherapy (B) Atezolizumab + chemotherapy (C) chemotherapy (D)Atezolizumab + Tiragolumab	TIGIT/CTLA-4	Hoffmann-La Roche
NCT03784326	II	December 31, 2024	Oesophageal or Gastroesophageal Junction adenocarcinoma - stage II/III, N=40	(A) Atezolizumab + chemotherapy (B) Atezolizumab + Tiragolumab + chemotherapy	TIGIT/CTLA-4	M.D. Anderson Cancer Center
NCT04543617	III	March 31, 2027	Unresectable OSCC, N=760	(A) Tiragolumab + Atezolizumab (B) Atezolizumab + placebo (C) double placebo	TIGIT/CTLA-4	Hoffmann-La Roche
NCT05743504	I/II	May 31, 2025	Resectable locally advanced OSCC, N=32	Tiragolumab and Atezolizumab with CCRT before surgery	TIGIT/CTLA-4	National Taiwan University Hospital
NCT05007106	II	February 22, 2027	Various solid tumours (including Oesophageal neoplasms), N=610	Pembrolizumab/Vibostolimab + chemotherapy	TIGIT/PD-1	Merck
NCT03829501	I/II	April, 2024	Various solid tumour (including EC), N=280	KY1044 monotherapy dose escalation and KY1044 and atezolizumab dose escalation	ICOS/PD-L1	Sanofi

Immune checkpoints play a major role in cancer immune evasion. Blocking the signals is one approach to promoting T-cell activity. Another approach is to inject checkpoint-deficient polyclonal T cells to replenish the effector population capable of targeting and killing tumour cells. Rapa Therapeutics is currently conducting a phase-I/II trial in several solid tumours, including oesophageal tumours, adding RAPA-201 cells to the standard of care chemotherapy protocols (NCT05144698). However, new targets are required as some ICI seem to decrease immune checkpoint expression on cells, which may contribute to developing ICI resistance (46). Trials attempt to assess the feasibility, safety and benefits from CLDN18.2 blockade (NCT03653507) (65), or RTK inhibitors added to PD-1 blockade (NCT04662710) (66).

## 4 Discussion and future perspectives

Despite tremendous results in other cancers, the mechanisms of immunotherapy response in OAC remain to be better understood in order to improve survival. There is growing evidence that the TME plays a critical role in treatment response and patient survival, particularly the infiltrating immune populations or stromal cells. OAC tumours are highly heterogeneous and surrounded by a largely immunosuppressive TME. However, individual study findings are conflicting due to the variability of the different study designs (sample collection, processing, used markers) or patient cohorts (demographics, stages). Efforts need to be directed towards better defining cell populations with multiple markers in large patient cohorts. Recent spatial technologies will provide additional details concerning the location and organisation of the immune infiltrate. Incorporating these insights with deep learning algorithms will lead to a better and more refined patient stratification. Grouping patients based on their tumour immune profiles appears to be a promising approach for advising the appropriate treatment or priming regimen for the tumour (8, 43). To date, patients with an immune enriched TME have better outcomes supporting the crucial role of the immune infiltrate. The second wave of blockade targeting LAG-3, TIM-3, TIGIT or ICOS will contribute to explore the anti-tumour immune response further.

## Author contributions

CB: Conceptualization, Visualization, Writing – original draft, Writing – review & editing. JL: Writing – review & editing. SB: Writing – review & editing. AB: Conceptualization, Writing – review & editing.
